# The effect of diabetes mellitus on organ dysfunction with sepsis: an epidemiological study

**DOI:** 10.1186/cc7717

**Published:** 2009-02-13

**Authors:** Annette M Esper, Marc Moss, Greg S Martin

**Affiliations:** 1 Division of Pulmonary, Allergy and Critical Care Medicine, Department of Medicine, Emory University, 201 Dowman Drive, Atlanta, Georgia 30322 USA; 2 Division of Pulmonary Sciences and Critical Care Medicine, Department of Medicine, University of Colorado at Denver and Health Sciences Center, 4200 E. Ninth Avenue, Denver, Colorado 80262 USA

## Abstract

**Introduction:**

Diabetes mellitus (DM) is one of the most common chronic co-morbid medical conditions in the USA and is frequently present in patients with sepsis. Previous studies reported that people with DM and severe sepsis are less likely to develop acute lung injury (ALI). We sought to determine whether organ dysfunction differed between people with and without DM and sepsis.

**Methods:**

Using the National Hospital Discharge Survey US, sepsis cases from 1979 to 2003 were integrated with DM prevalence from the Centers for Disease Control and Prevention (CDC) Diabetes Surveillance System.

**Results:**

During the study period 930 million acute-care hospitalisations and 14.3 million people with DM were identified. Sepsis occurred in 12.5 million hospitalisations and DM was present in 17% of patients with sepsis. In the population, acute respiratory failure was the most common organ dysfunction (13%) followed by acute renal failure (6%). People with DM were less likely to develop acute respiratory failure (9% vs. 14%, p < 0.05) and more likely to develop acute renal failure (13% vs. 7%, p < 0.05). Of people with DM and sepsis, 27% had a respiratory source of infection compared with 34% in people with no DM (p < 0.05). Among patients with a pulmonary source of sepsis, 16% of those with DM and 23% of those with no DM developed acute respiratory failure (p < 0.05); in non-pulmonary sepsis acute respiratory failure occurred in 6% of people with DM and 10% in those with no DM (p < 0.05).

**Conclusions:**

In sepsis, people with diabetes are less likely to develop acute respiratory failure, irrespective of source of infection. Future studies should determine the relationship of these findings to reduced risk of ALI in people with DM and causative mechanisms.

## Introduction

Sepsis is a common disease that continues to increase in incidence in the USA [1]. Severe sepsis, sepsis associated with acute organ system dysfunction, is frequently encountered in the intensive care unit (ICU) population and is associated with a high morbidity and mortality [2]. Of the disorders commonly associated with acute lung injury (ALI), sepsis carries the highest risk of progression at about 40% [3,4]. Specific risk factors, including age and chronic co-morbid medical conditions, such as chronic liver disease, HIV infection and cancer, have been identified that predispose patients to sepsis or severe sepsis [5-9]. The ability of chronic co-morbid medical conditions, such as diabetes mellitus (DM), to influence the risk of sepsis or sepsis-related organ dysfunction remains unclear.

DM is one of the most common diseases in the USA, with statistics from the Centers for Disease Control and Prevention (CDC) reporting that in 2005 almost 21 million people in the USA (7% of the population) have DM. The prevalence of DM is rising [10] and from 2002 to 2005, 2.6 million individuals have been newly diagnosed with DM. Patients with DM are at increased risk of developing infections and are frequently part of both epidemiological studies and clinical trials in critically ill patients. For example, two studies have suggested that patients with DM and septic shock are less likely to develop ALI [11,12]. This may be due to differences in the inflammatory response between people with and without DM. However, at this time the aetiology for this association remains unclear, and may represent differential risk for organ dysfunction as a whole. In order to further understand differences between critically ill patients with and without DM, we sought to identify differences in organ dysfunction between patients with and without DM and sepsis. Accurate identification of populations at risk for acute organ dysfunction is crucial to improve our understanding of the mechanisms involved and to develop novel therapies for these patients.

## Materials and methods

### Dataset

The National Center for Health Statistics has conducted the National Hospital Discharge Survey (NHDS) continuously since 1965 [13]. Since 1979, the NHDS has conformed to the guidelines of the Uniform Hospital Discharge Data Set for consistency of reporting in records. The NHDS is composed of a sample of all nonfederal acute care hospitals in the USA, including about 500 hospitals. Discharge records from inpatients are surveyed from each hospital, representing about 1% of all hospitalisations in the USA. The database includes patient-specific information such as age, sex, self-reported racial category, seven diagnostic and four procedural codes (from the Clinical Modification of the International Classification of Diseases, 9^th ^Revision (ICD-9-CM)), sources of payment and discharge disposition. DM prevalence from 1979 to 2003 was obtained from the CDC Diabetes Surveillance System which collects, analyses and disseminates data on DM and its complications [14].

### Identification of cases

Cases of patients with sepsis were identified from discharge records in the NHDS during the 25-year period from 1979 to 2003 that included an ICD-9-CM code for sepsis as previously validated [1,15]: 038.*x *(septicaemia), 790.7 (bacteraemia), 117.9 (disseminated fungal infection), 112.5 (systemic candidiasis) and 112.81 (disseminated fungal endocarditis). Type of infection refers to the causative organism for sepsis; source of infection refers to the anatomical site of infection. Type and source of infection, DM and acute organ dysfunction were identified using ICD-9 groupings, as previously published. Source of infection: Respiratory 010.0 to 011.9, genitourinary 098.17, gastrointestinal 001.9 to 009.9, bone/joint 730.9, skin/soft tissue 003.24, central nervous system (CNS) 013, cardiovascular 036.45 to 036.43; DM 250.x; organ dysfunction: respiratory 96.7× to ventilator management, 518.x to acute respiratory failure, acute respiratory distress syndrome (ARDS), ARDS after shock or trauma, cardiovascular 458.x, 785.5, renal 39.95, 580.x, 584.x, hepatic 570, haematological 287.4, 286.9, metabolic 276.2, CNS 780.01, 780.09, 348.x.

Chronic co-morbid medical conditions were also cumulatively quantified by an established co-morbidity index (Charlson-Deyo score) [16-18]. Other outcome variables such as mortality, length of stay and discharge status were collected. All data collected represent data available during hospitalisation, therefore long-term outcome data is not available. This project was exempt from the requirement for informed consent according to federal regulations of human subjects protection 45 CFR § 46.101(b). The Emory Institutional Review Board approved the study as exempt from the requirement for consent.

### Statistical analysis

All estimates are presented according to accepted guidelines for the accuracy of NHDS data, restricting use to absolute, unweighted samples of more than 60 patients with relative standard error (RSE) measures of less than 30% for data analysis. The RSE was calculated as a first-order Taylor-series approximation, as outlined in the RSE tables of the 2000 NHDS documentation. The standard error was calculated by multiplying the RSE by the estimated incidence or mortality rate, and 95% confidence intervals (CI) were calculated from these standard errors with the use of Excel software (Microsoft Corporation, Redmond, Washington). Data for continuous variables were compared by analysis of variance and data for categorical variables were compared by the chi-squared test, with the use of SAS software (SAS 9.1 for Windows; SAS Institute, Cary, North Carolina). When stated race was missing for a given observation (ranging from 1 to 20% for any given year), these persons were excluded from the calculations of race specific rates but were included in all other calculations of rates. An *a priori *stratified analysis between pulmonary and non-pulmonary sources of sepsis was conducted to differentiate the risk of acute respiratory failure in patients with a pulmonary source of infection. Differences were considered significant when the 95% CIs did not overlap and/or when two-sided p-values were less than 0.05.

## Results

Using the NHDS, from the years 1979 to 2003, there were 12.5 million cases of sepsis identified and DM was present in 17% (2,070,459) of the cases. Based on the CDC Diabetes Surveillance System, the number of persons with DM in the USA increased from 5,762,000 persons in 1980 to 14,275,000 in 2003. Among general hospitalised patients the frequency of DM increased from 5.2% in 1979 to 13.3% in 2003. The frequency of DM among septic patients increased from 11% (17,249 cases) in 1979 to 18% (122,824 cases) in 2003.

### Demographics and causes of sepsis

Demographic data for populations with and without DM and sepsis are shown in Table 1. Forty-three percent (892,230) of people with DM and sepsis were male and 57% (1,178,229) were female (p < 0.001). Among the patients with sepsis and DM, 64% were white, 17% black and 5% other race. The mean number of co-morbidities was greater in people with DM and sepsis (2.07) compared with people with sepsis and no DM (0.88, p < 0.0001).

**Table 1:** Demographic characteristics of patients with sepsis from 1979 to 2003.

	**Patients with diabetes**	**Patients with no diabetes**
**Number of patients (n)**	2,070,459	10,430,000
**Age (mean, years)**	68	59
**Race**		
**Caucasian**	64%	67%
**Black**	17%	14%
**Other**	5%	4%
**Unknown**	14%	15%
**Gender**		
**Male (%)**	43%	49%
**Female (%)**	57%	51%
**Pathogens (%)**		
**Gram negatives**	25%	22%
**Gram positives**	29%	28%
**Anaerobic**	1%	1%
**Fungal**	2.4%	2.2%
**Unknown**	43%	49%
**Number of co-morbidities (mean)**	2.07	0.88
**Number of dysfunctional organ systems (mean)**	0.36	0.41
**Mean hospital length of stay (days)**	13	14
**Case fatality (%)**	18.5%	20.6%

**Figure 1:**
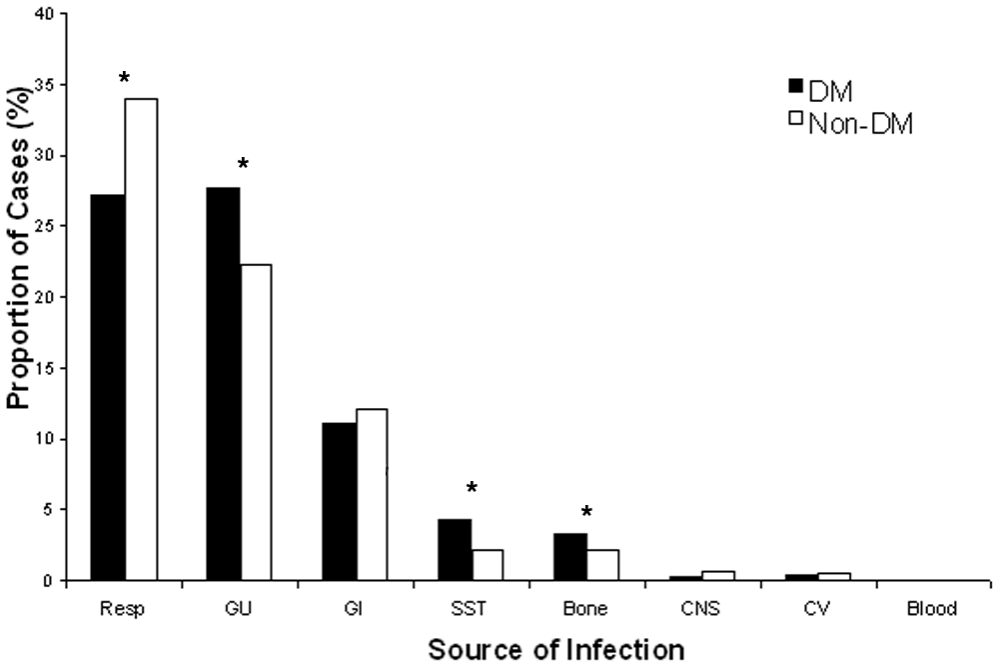
Frequency of sepsis cases. Frequency of sepsis cases among patients with diabetes mellitus (DM) and those with no diabetes mellitus (non-DM) with a source of infection identified. CV = cardiovascular; GI = gastrointestinal; GU = genitourinal; Resp = respiratory; SST = skin and soft tissue. * p < 0.05.

Figure 1 represents the sources of infections in patients with and without DM and sepsis. A respiratory source of infection was present in 27% of patients with DM and sepsis compared with 34% in patients with no DM (p < 0.05). People with DM had an increased frequency of genitourinary (GU; 28% vs. 22%), skin soft tissue (4% vs. 2%) and bone (3% vs. 2%) infections in comparison to people with no DM (p < 0.05).

### Organ dysfunction

In the overall population of patients with sepsis, 13% developed acute respiratory failure, 8% acute renal failure, 4% cardiovascular failure and 6% developed other organ dysfunctions. People with DM and sepsis were more likely to develop acute renal failure compared with people with no DM (13% vs. 7%, p < 0.05) and are less likely to develop acute respiratory failure (9% vs. 14%, p < 0.05). There were no differences in the occurrence of other organ dysfunctions between the two groups or in the overall mean number of organ dysfunctions.

**Figure 2:**
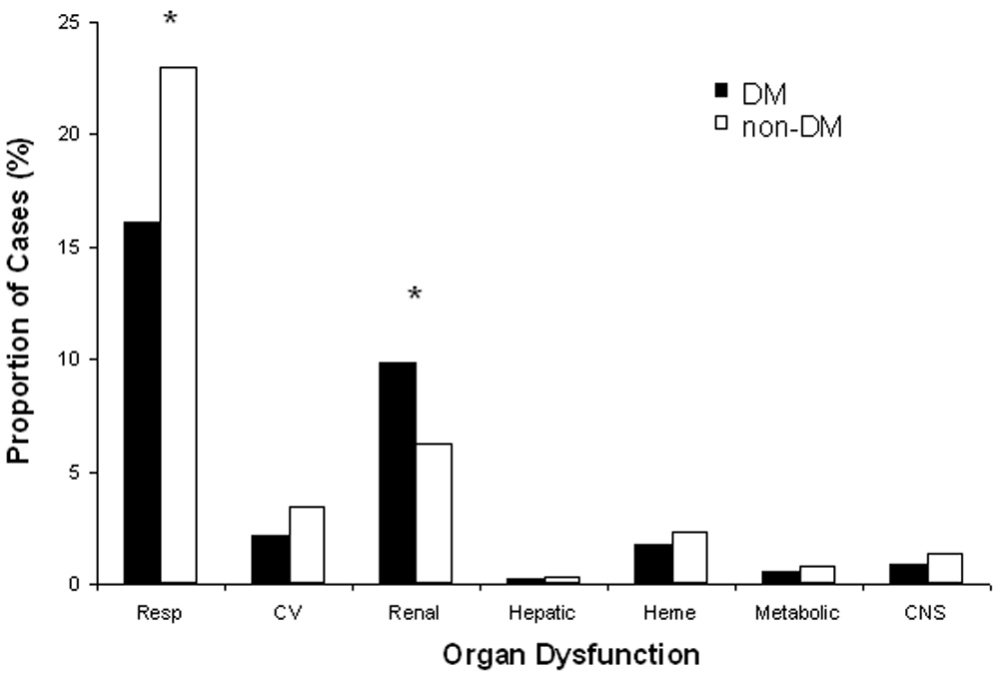
Frequency of acute organ dysfunction. Frequency of acute organ dysfunction in patients with diabetes mellitus (DM) and those with no diabetes mellitus (non-DM) with a respiratory source of sepsis. CV = cardiovascular; Heme = haematological; Resp = respiratory. * p < 0.05.

**Figure 3:**
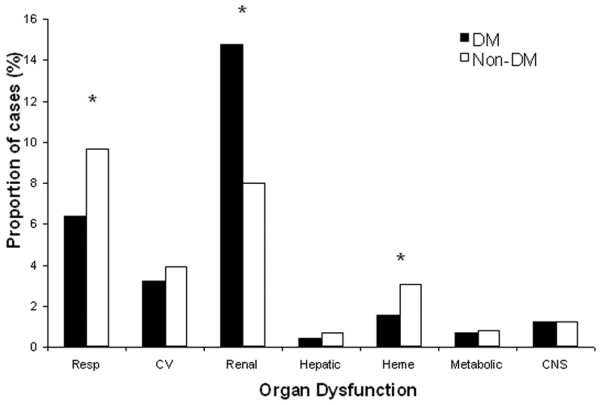
Frequency of organ dysfunction. Frequency of organ dysfunction in patients with diabetes mellitus (DM) and those with no diabetes mellitus (non-DM) with a non-respiratory source of sepsis. CV = cardiovascular; Heme = haematological; Resp = respiratory. * p < 0.05.

To account for differences between people with and without DM, the frequency and type of organ dysfunction was examined within strata of infection sources. Among patients with respiratory source of sepsis, 16% of those with DM developed acute respiratory failure compared with 23% in people with no DM (p < 0.05). Among patients with a non-pulmonary source of sepsis, those with DM were still less likely to develop acute respiratory failure when compared with those with no DM (6% vs. 10%, p < 0.05). People with DM and sepsis were more likely to develop acute renal failure than those with no DM irrespective of the source of infection (10% vs. 6% for pulmonary sepsis, 14% vs. 8% for non-pulmonary sepsis; both p < 0.05). When a GU source of infection was compared with a non-GU source of infection, people with DM and sepsis were still more likely to develop acute renal failure than those with no DM. Among people with DM and sepsis, 46% with a non-GU source of infection developed acute renal failure, compared with 44% with a GU source of infection. The only other significant difference in organ dysfunction was observed in non-pulmonary sepsis: haematological failure occurred in 1.6% of patients with DM and in 3.1% of those with no DM (p < 0.05) (Figures 2 and 3).

### Outcomes

Among patients with sepsis, the case-fatality rate was lower for those with DM at 18.5% versus 20.6% in those with no DM (p < 0.05). No other significant differences were found in case fatality with respect to source of infection except for GU sepsis at 9% for those with DM vs. 12% in patients with no DM (p < 0.05). People with DM and sepsis who developed acute respiratory failure had a case fatality rate of 52% versus 48% in those with no DM (p = NS).

### Discharge status

Over the study period, the hospital length of stay for people with DM was 12.8 days versus 14.1 days in those with no DM (p < 0.001). Discharge status during the study period was different between patients with and without diabetes: patients with no DM were more likely to be discharged home (65% vs. 58%, respectively, p < 0.05) whereas patients with DM were more likely to be discharged to an outside health care facility (32% vs. 28%, respectively, p < 0.05).

## Discussion

The current epidemiological study allows us to further characterise the impact of DM on the development of organ dysfunction among patients with sepsis. When compared with patients with severe sepsis and no DM, people with DM are less likely to develop acute respiratory failure. The lower risk of acute respiratory failure among patients with severe sepsis and DM was irrespective of whether the primary source of infection was pulmonary or non-pulmonary. With respect to other organ dysfunctions, people with DM were more likely to develop acute renal failure. The presence of a GU source of infection did not affect the development of acute renal failure among those with DM. The decrease in the frequency of respiratory failure in people with DM was associated with a significant difference in case fatality.

These data are consistent with previous observations made by our group in a study evaluating the impact of DM on the development of ARDS in patients with septic shock [11]. In that prospective multi-centre ICU study, 28% of the patients with septic shock had a history of DM. Patients in the ICU with no DM were more likely to have pneumonia, urinary tract and abdominal infections. Only 25% of patients with DM developed ARDS compared with 47% of those with no DM (p = 0.03, relative risk = 0.53, 95% CI = 0.28 to 0.98). In a multivariable model, the protective association between DM and the development of ARDS remained significant. Our novel observation was confirmed in another prospective cohort study of 688 heterogeneous patients in the ICU [12]. After multivariate adjustment, DM was again associated with a decreased risk of ARDS, with a similar odds ratio of 0.58 (95% CI = 0.36 to 0.92). In agreement with the current study, the above data suggest that people with DM and a variety of other conditions are less likely to develop both acute respiratory failure and ARDS.

Our present study has limitations related to the use of hospital administrative data. Although the use of ICD-9 codes to identify specific medical conditions is not ideal, it has been validated for sepsis as having a positive predictive value of 88.9% and a sensitivity of 87.7% [1,15]. Individual patient-level data, such as medications, haemoglobin A_1C _levels and glucose levels would be difficult to obtain from such data sets. The study is further limited by the lack of data on severity of organ dysfunction, which may have implications on other outcomes. Discrimination between patients with type 1 and type 2 DM may also provide clues to mechanisms for differential organ dysfunction. However, the large sample size of patients obtained from utilising a national data base may offset some of these limitations.

The mechanisms responsible for this epidemiological association between DM and ARDS are unclear. The effect of DM on the immune system and inflammatory response is thought to play a role [19], and perhaps a blunted inflammatory response effects the development of organ dysfunction in sepsis. Possible mechanisms of protection in patients with DM may be impaired neutrophil function or altered neutrophil-endothelial interactions [20,21]. Obtaining data on specific inflammatory markers that may play a role in the differences in response to an infectious insult may clarify the association as well.

Hyperglycaemia may be another factor that influences the development of ARDS. In our previous prospective study, there was a trend towards a lower incidence of ARDS in hyperglycemic patients with no DM; however, this was based on admission glucose values. This effect may be better understood if haemoglobin A_1C _levels were available as a marker of previous glycaemic control, in addition to serial glucose levels during the patient's stay in the ICU. Another possible explanation for the association between DM and the risk of ARDS may relate to increased medical care among patients with DM. Patients with DM may be hospitalised earlier than those with no DM in the course of their illness because they learn to be aware of specific signs of infection. Information on timing of presentation and onset of symptoms, however, may be difficult to obtain in many patients.

Pharmacological aspects of DM care may also influence the development of organ dysfunction, because many medications administered to patients with DM, including insulin and thiazolidinediones (TZDs), are known to have anti-inflammatory effects in addition to lowering blood glucose. Although the role of intensive insulin therapy in patients with severe sepsis remains uncertain, insulin may have other beneficial effects in this patient population. A key feature of ARDS is the systemic production of pro-inflammatory mediators and cytokines, such as tumour necrosis factor (TNF) α, interleukin (IL) 1β, IL-6 and IL-8, which have been found in the bronchoalveolar lavage fluid and plasma of patients with ARDS; and elevated concentrations have been associated with an unfavourable outcome [22-24]. A critical mediator of this inflammatory cascade is the transcriptional regulatory factor nuclear factor (NF) κB, which may be suppressed by insulin administration. Insulin administration to animals challenged with lipopolysaccharide inhibits TNFα production in a dose-dependent manner [25] and prevents the development of ALI [26]. Similarly, TZDs may modulate the inflammatory response through the peroxisome-proliferator-activated receptor gamma and at the transcriptional level through inhibition of NF-κB activity [27-31]. Further investigations on the role of insulin and TZDs on the inflammatory response are necessary to identify a possible mechanism for affecting the development of ALI.

## Conclusions

This study confirms previous observations that a history of DM is associated with a lower incidence of acute respiratory failure in patients with severe sepsis. The information obtained moves us a step closer to better understanding the pathogenesis of sepsis and sepsis-related organ dysfunction, such as ALI. Identifying conditions that have an effect on the propensity to develop organ dysfunction in sepsis will allow for the expansion of studies on interventions for this disease. Further prospective data need to be collected in this cohort of patients to identify the factors that contribute to this protective effect of DM.

## Key messages

• Patients with DM and severe sepsis are less likely to develop acute respiratory failure than patients with no DM, irrespective of source of infection.

• Patients with DM and severe sepsis are more likely to develop acute renal failure than patients with no DM.

• The decreased frequency of acute respiratory failure in patients with DM and severe sepsis did not translate into a significant difference in case fatality.

## Abbreviations

ALI: acute lung injury; ARDS: acute respiratory distress syndrome; CDC: Centers for Disease Control and Prevention; CNS: central nervous system; DM: diabetes mellitus; GU: genitourinary; ICD-9-CM: Clinical Modification of the International Classification of Diseases, 9^th ^Revision; IL: interleukin; NF: nuclear factor; NHDS: National Hospital Discharge Survey; RSE: relative standard error; TNF: tumour necrosis factor; TZD: thiazolidinediones.

## Competing interests

The authors declare that they have no competing interests.

## Authors' contributions

AE carried out the main analysis and interpretation of the data, in addition to preparing the manuscript. MM and GM both contributed to the design of the study, interpretation of the data, statistical analysis and manuscript revision. All authors read and approved the final manuscript.
